# Design and Analysis of MEMS Linear Phased Array

**DOI:** 10.3390/mi7010008

**Published:** 2016-01-15

**Authors:** Guoxiang Fan, Junhong Li, Chenghao Wang

**Affiliations:** State Key Laboratory of Acoustics, Chinese Academy of Sciences, Beijing 100190, China; fanguoxiang13@mails.ucas.ac.cn (G.F.); chwang@mail.ioa.ac.cn (C.W.)

**Keywords:** MEMS, “multi-cell” element, linear phased array, three-dimensional directivity

## Abstract

A structure of micro-electro-mechanical system (MEMS) linear phased array based on “multi-cell” element is designed to increase radiation sound pressure of transducer working in bending vibration mode at high frequency. In order to more accurately predict the resonant frequency of an element, the theoretical analysis of the dynamic equation of a fixed rectangular composite plate and finite element method simulation are adopted. The effects of the parameters both in the lateral and elevation direction on the three-dimensional beam directivity characteristics are comprehensively analyzed. The key parameters in the analysis include the “cell” number of element, “cell” size, “inter-cell” spacing and the number of elements, element width. The simulation results show that optimizing the linear array parameters both in the lateral and elevation direction can greatly improve the three-dimensional beam focusing for MEMS linear phased array, which is obviously different from the traditional linear array.

## 1. Introduction

Ultrasonic transducers and their array have an important application on non-destructive testing and medical acoustic imaging. Compared with current transducers, based on lead zirconate titanate (PbZr*_x_*Ti_1−*x*_O_3_, PZT) bulk materials, piezoelectric micro-machined ultrasonic transducer (pMUT) fabricated by piezoelectric film deposition and micro-electro-mechanical system (MEMS) technology has great advantages in integration, which can structure system-in-package (SIP) and even system-on-chip (SOC) technologies. Moreover, the technical challenge of three-dimensional acoustic imaging is the fabrication of two-dimensional array transducers with many elements, high element density and small element size. Lapping and dicing bulk materials will become more difficult with traditional technologies of transducer fabrication. Furthermore, the high frequency MEMS transducers with good resolution are easier to achieve than those prepared by traditional fabrication technologies, and the vibration of MEMS piezoelectric transducer is mostly a bending vibration with the advantages of high sensitivity, wide bandwidth, flexible design, *etc.* [1]. As an important pMUT array, a MEMS linear phased array has been developed [2,3,4,5].

The pMUT mostly works in the bending vibration mode [1,6,7], and the area of vibration film decreases as the resonant frequency increases. As a result, the sound pressure of the radiation becomes weak when the array works at high frequency. For a transducer, the working frequency is an important parameter and should be predicted. There are several analysis methods to predict the resonant frequency of an element [8,9,10], and the results are obviously different. Thus, the right way to more accurately predict the resonant frequency needs to be found.

The structure optimization of a transducer array has been carried out to improve the radiated sound field directivity [11,12,13,14,15,16,17]. Steinberg studied the focusing properties of uniform linear array composed of many point sources, ignoring the array size [11]. Wooh *et al.* studied the linear array focusing effect, which the element length is infinite or the element length is much larger than the element width [14,15]. Actually, the element of phased array has certain size, and the array could have good effect of the spatial focusing when the main lobe of its beam has the three-dimensional structure of the slender spindle type in steering angle direction. Wooh *et al.* studied the influence of the elevation dimension on the steering performance of the traditional ultrasonic linear phased array [18]. They found sideleaking energy exists for array with short element length and increases when the ratio of length to width decreases. In fact, the energy leakage caused by the non-azimuthal plane can decrease the ratio of signal to noise in the azimuthal plane. Compared with traditional one-dimensional linear array, the sideleaking energy is more serious in the MEMS transducer array for smaller ratio of length to width. Therefore, the study on the influence of the MEMS phased array parameters in the elevation direction on the three dimensional directivity of the acoustic beam is very necessary. However, the theory and experiment on above study have never been carried out.

In our paper, the MEMS linear phased array working in bending vibration mode at high frequency is designed, which is based on the “multi-cell” elements, and each element is made of many “cells” by parallel connection. The dynamic equation of theoretical analysis and ANSYS software simulation (the finite element analysis) are used to more accurately predict the array resonant frequency. Through analyzing the three-dimensional focusing characteristics, the effects of the structure parameters both in the lateral and elevation direction on the directivity are studied and the parameters include the “cell” number of element, the “cell” size, the “inter-cell” spacing and number of elements and element width. Finally, the optimization results are simulated using MATLAB tools.

## 2. MEMS Linear Phased Array Element Structure

**Figure 1:**
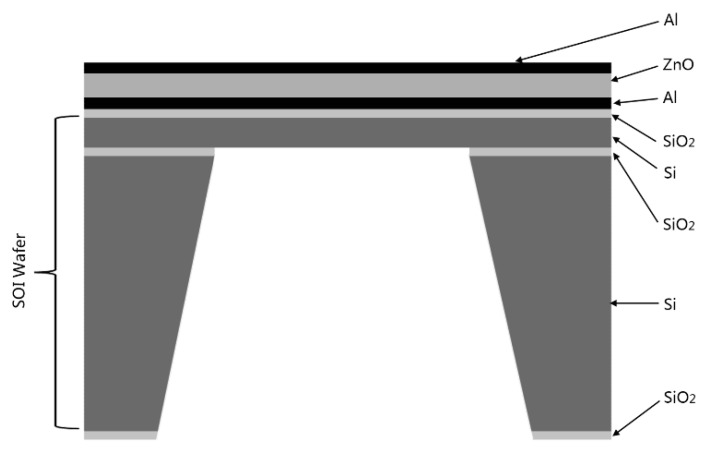
Structure of MEMS piezoelectric transducer.

Determination of the array resonant frequency has a significant influence on the array optimization. It depends on the structure of composite plate, the thickness of each film, the properties of material, *etc*. The vibration film of MEMS piezoelectric transducer is composed of the piezoelectric layer and the supporting layer. In order to reduce the complexity of the fabrication process, the square vibration film of transducer is adopted. In this paper, ZnO film is used as the piezoelectric material, SiO_2_/Si film as the supporting layer, and Al film as the upper and lower electrode. Thus, the vibration membrane is Al/ZnO/Al/SiO_2_/Si composite structure (see Figure 1). Composite membrane can be considered as a square diaphragm that is fixed on all edge sides.

The transducer’s transmit and receive performance are best when it works at the resonant frequency. There are several methods to predict the resonant frequency of the vibration films. Using the method of the theoretical analysis of the dynamic equation of a fixed rectangular composite plate, the resonant frequency of the transducer is calculated as [8]:

(1) $$f_{11}=\frac{17.994}{\pi a^{2}}\sqrt{\frac{D}{\rho h}}$$

where *D* is bending rigidity, ρ is average density, and *a* and *h* are length and thickness of the diaphragm, respectively. The physical parameters and material properties of the transducer used in theory analysis are summarized in Table 1 and Table 2, respectively. The resonant frequency of transducer is calculated as *f*_11_ = 3 MHz.

Yamashita *et al.* used the following expression to calculate the resonant frequency [9]:

(2) $$f_{r}=\frac{35.99}{2\pi }\sqrt{\frac{K}{(1-\nu^{2})\rho ta^{4}}}$$

where *K* is bending rigidity, ν is Poisson’s ratio, ρ is average density, and *a* and *t* are length and thickness of the diaphragm, respectively. Using the parameters in Table 1 and Table 2, the resonant frequency of transducer is calculated as *f*_r_ = 3.23 MHz.

Dausch *et al.* used the following expression to calculate the resonant frequency [10]:

(3) $$f_{0}=1.028\frac{t}{l^{2}}\sqrt{\frac{E}{\rho }}$$

where *E* is average Young’s modulus, ρ is average density, and *l* and *t* are length and thickness of the diaphragm, respectively. Using the parameters in Table 1 and Table 2, the resonant frequency of transducer is calculated as *f*_0_ = 1.75 MHz.

**Table 1:** Structure parameters of the multi-membrane.

Thickness of Si	Thickness of SiO_2_	Thickness of ZnO	Length of Membrane
8 μm	0.2 μm	6 μm	228 μm

**Table 2:** Materials properties of transducer.

Material	Young’s Modulus (GPa)	Density (10^3^ kg/m^3^)	Poisson Ratio
Si	167	2.33	0.28
SiO_2_	72	2.30	0.16
ZnO	120	5.68	0.446

**Figure 2:**
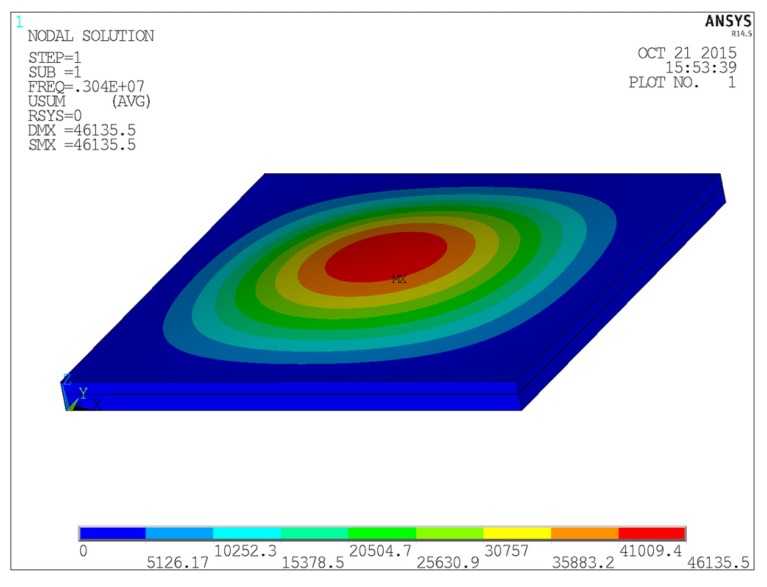
Amplitude distribution of first order resonant mode of vibration film.

The resonant frequency of the transducer with the same structure parameters is also analyzed by finite element method and the result is compared with Equations (1)–(3). It is the piston type vibration mode of the first order mode. The displacement of each point on the vibrating plate is same in the Z-direction. The first order mode is shown in Figure 2. The result of ANSYS software simulation is consistent with the theoretical analysis of the dynamic equation (Equation (1)) of a fixed rectangular composite plate. The simulated resonant frequency is 3.04 MHZ which is very close to the result of Equation (1) 3 MHz and is obviously different from the result of Equation (2) 3.23 MHz and the result of Equation (3) 1.75 MHz. In our work, the theoretical analysis of the dynamic equation (Equation (1)) of a fixed rectangular composite plate and ANSYS software simulation are adopted to more accurately predict the array resonant frequency.

**Figure 3:**
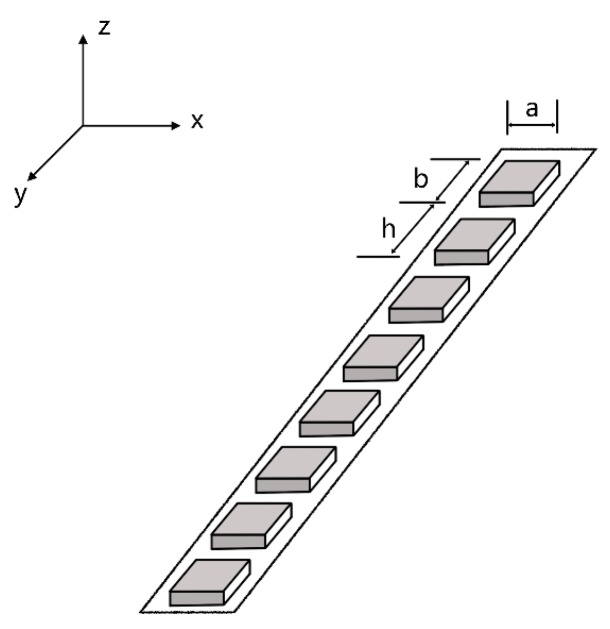
Structure of one element (“multi-cell” combined by parallel connection). *a*, *b* and *h* represent element (“cell”) width, “cell” length, and “inter-cell” spacing, respectively.

**Figure 4:**
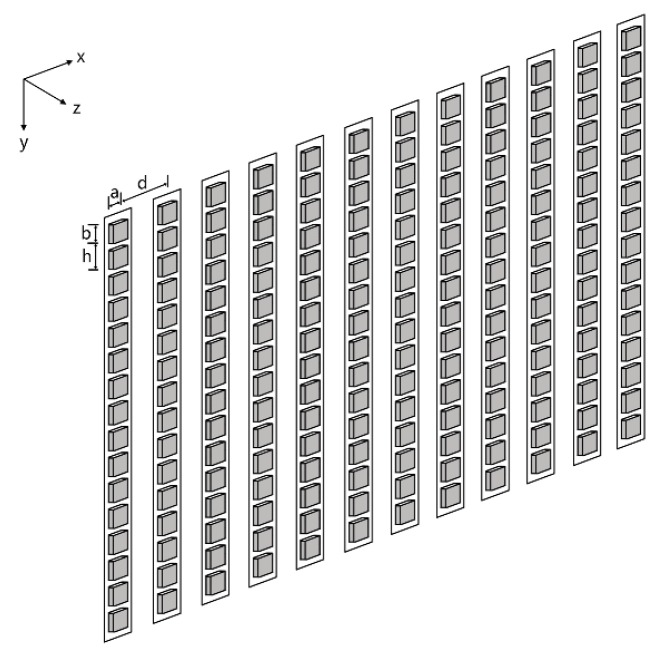
Schematic diagram of linear phased array. *a*, *b*, *d* and *h* represent element (“cell”) width, “cell” length, element width and “inter-cell” spacing, respectively.

It can be seen that array elements can cause a lower resonant frequency whose vibration films design is too long. In order to increase radiation sound pressure of the MEMS linear phased array working in bending vibration mode at high working frequency, we designed an array structure based on “multi-cell” elements. The single “cell” is a piezoelectric transducer whose vibration membrane is square. Many “cells” form the element of linear phased array by parallel connection. *h*, *a* and *b* (*a* is equal to *b* when it is a square “cell”) are the “inter-cell” spacing, the “cell” width, and “cell” length, respectively (see Figure 3). The *N* elements are uniformly arranged in *x-y* plane. When it works, each array element is individually activated. The array is shown in Figure 4.

## 3. MEMS Linear Phased Array Beam Directivity

The directivity function of the complex array is generally simplified as a combination of the directivity function of some simple structure ones, which is commonly carried out using product theorems. Using product theorems, the complex array directivity function can be obtained by taking the product of the directivity function of the sub-array.

For the MEMS linear array shown in Figure 4, the radiated sound field directivity function is equal to the product of a single element directivity function and a linear array directivity function composed of *N* point source in each element center.

1. The directivity of a single element

**Figure 5:**
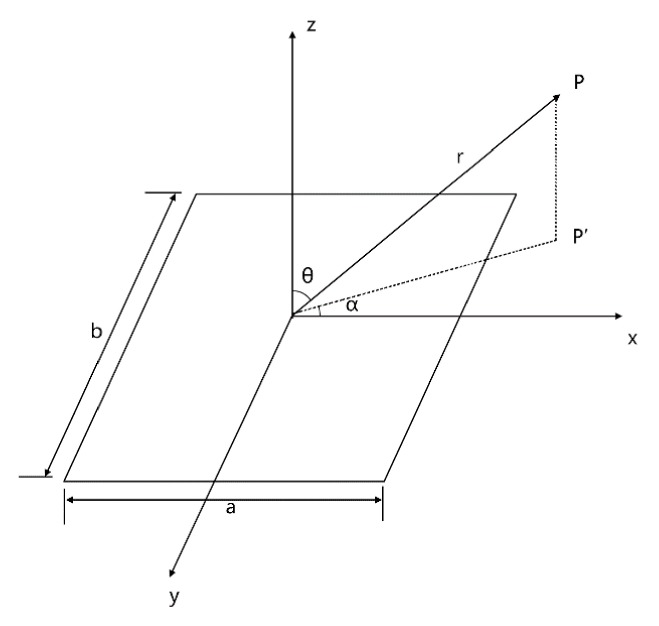
A single rectangular plunger.

For a single rectangular plunger (see Figure 5), the sound pressure *P* in the sound field is given by:

(4) $$p=j\frac{kcu_{a}\rho_{0}}{2r\pi }ab\frac{\sin\left(\frac{kb\sin\theta \sin\alpha }{2}\right)}{\frac{kb\sin\theta \sin\alpha }{2}}\cdot \frac{\sin\left(\frac{ka\sin\theta \cos\alpha }{2}\right)}{\frac{ka\sin\theta \cos\alpha }{2}}\cdot \exp\left[j\left(\omega t-kr\right)\right]$$

where k is the wavenumber, $\rho_{0}$ is the medium density, $u_{a}$ is the array element vibration velocity, r is the distance from the rectangular plunger to point P(θ,α), and a and b are rectangular plunger length and width, respectively.

The single array element is considered as M rectangular plungers in the *y*-axis (see Figure 3), and the distance from the *i*th “cell” unit to point P(θ,α) can be expressed as:

(5) $$r_{i}=\sqrt{r^{2}+{\left(ih\right)}^{2}+2r\cdot ih\cdot \sin\theta \sin\alpha }$$

By transformation of Taylor’s first order expansion, Equation (5) becomes:

(6) $$r_{i}=r+ih\cdot \sin\theta \sin\alpha$$

Because the “cells” of single array element are activated at the same time, the sound waves are emitted by the same amplitude and phase. According to Huygens principle and Equations (4) and (6), the total sound pressure of the array elements is given by:

(7) $$\begin{array}{ccc} p\left(r,\alpha ,\theta ,t\right) & = & \sum_{i=0}^{M-1}p_{i}\left(r,\alpha ,\theta ,t\right)\\ & = & \sum_{i=0}^{M-1}j\frac{kcu_{a}\rho_{0}}{2r\pi }ab\frac{\sin\left(\frac{kb\sin\theta \sin\alpha }{2}\right)}{\frac{kb\sin\theta \sin\alpha }{2}}\cdot \frac{\sin\left(\frac{ka\sin\theta \cos\alpha }{2}\right)}{\frac{ka\sin\theta \cos\alpha }{2}}\cdot \exp\left[j\left(\omega t-kr_{i}\right)\right]\\ & = & A\cdot \frac{\sin\left(\frac{kb\sin\theta \sin\alpha }{2}\right)}{\frac{kb\sin\theta \sin\alpha }{2}}\cdot \frac{\sin\left(\frac{ka\sin\theta \cos\alpha }{2}\right)}{\frac{ka\sin\theta \cos\alpha }{2}}\cdot \frac{\sin\left(\frac{kh\sin\theta \sin\alpha }{2}M\right)}{M\sin\left(\frac{kh\sin\theta \sin\alpha }{2}\right)} \end{array}$$

where

(8) $$A=j\frac{kcu_{a}\rho_{0}}{2r\pi }abM\cdot \exp\left[-j\left(M-1\right)\frac{kh\sin\theta \sin\alpha }{2}\right]\cdot \exp\left[j\left(\omega t-kr\right)\right]$$

According to the radiation principle of the acoustic field and the definition of the directivity function, the directivity function of the array element is given by:

(9) $$D(\alpha ,\theta ,\omega )=\left|\frac{\sin\left(\frac{\pi a}{\lambda }\cos\alpha \sin\theta \right)}{\frac{\pi a}{\lambda }\cos\alpha \sin\theta }\cdot \frac{\sin\left(\frac{\pi b}{\lambda }\sin\alpha \sin\theta \right)}{\frac{\pi b}{\lambda }\sin\alpha \sin\theta }\cdot \frac{\sin\left(M\frac{\pi h}{\lambda }\sin\alpha \sin\theta \right)}{M\sin\left(\frac{\pi h}{\lambda }\sin\alpha \sin\theta \right)}\right|$$

2. The uniform linear array directivity

Considering the uniform linear array composed of *N* point sources, each array element have the same resonant frequency and vibration amplitude, but have the different phase. The sound pressure normalized directivity function is given by:

(10) $$D\left(\alpha ,\theta ,\omega \right)=\left|\frac{\sin\left[N\frac{\pi d}{\lambda }\left(\cos\alpha \sin\theta -\cos\alpha_{0}\sin\theta_{0}\right)\right]}{N\sin\left[\frac{\pi d}{\lambda }\left(\cos\alpha \sin\theta -\cos\alpha_{0}\sin\theta_{0}\right)\right]}\right|$$

Multiplication manipulation using Equations (9) and (10) leads to the following expression for the sound pressure directivity function of the array D(α,θ,ω):

(11) $$D\left(\alpha ,\theta ,\omega \right)=|\frac{\sin\left[N\frac{\pi d}{\lambda }\left(\cos\alpha \sin\theta -\cos\alpha_{0}\sin\theta_{0}\right)\right]}{N\sin\left[\frac{\pi d}{\lambda }\left(\cos\alpha \sin\theta -\cos\alpha_{0}\sin\theta_{0}\right)\right]}\cdot \frac{\sin\left(\frac{\pi a}{\lambda }\cos\alpha \sin\theta \right)}{\frac{\pi a}{\lambda }\cos\alpha \sin\theta }\cdot \frac{\sin\left(\frac{\pi b}{\lambda }\sin\alpha \sin\theta \right)}{\frac{\pi b}{\lambda }\sin\alpha \sin\theta }\cdot \frac{\sin\left(M\frac{\pi h}{\lambda }\sin\alpha \sin\theta \right)}{M\sin\left(\frac{\pi h}{\lambda }\sin\alpha \sin\theta \right)}|$$

## 4. Optimization and Simulation

The sound beam that the phased array radiated to the three-dimensional space will produce a relative maximum amplitude of the sound pressure in a steering angle direction. If the designed parameters of the phased array are not optimized, the radiation of the sound beam will tend to produce the grating lobes and side lobes around the main lobe. The existence of the grating lobes and the side lobes means the sound waves propagate in other directions, which causes the “leakage” of the beam energy and affects the signal to noise ratio of the system. Thus, the array parameters should be optimized by minimizing the main lobe width, eliminating grating lobes, and suppressing side lobes as much as possible.

For the MEMS linear array, we designed “cells” of a single array element that will activate simultaneously, and each element is activated by different time series delay. This kind of array structure is generally produce grating lobes and side lobes in the two main directions, the elevation direction and the lateral direction. Thus, the goal of optimization can be achieved by determining the array parameters in these two directions.

### 4.1. Optimization in the Elevation Direction (y-z Plane, α = α_0_ = 90°)

#### 4.1.1. Main Lobe

Main lobe width is the distance between the intersection points of the main lobe and the θ axis in the plane pattern, so the width can be obtained by letting the directivity function *D*(θ) = 0.

Considering α = α_0_ = 90*°*, the beam directivity function of Equation (11) can be simplified to:

(12) $$D\left(\frac{\pi }{2},\theta ,\omega \right)=D_{1}\left(\theta \right)\cdot D_{2}\left(\theta \right)$$

where *D*_1_(θ) is given by:

(13) $$D_{1}\left(\theta \right)=\left|\frac{\sin\left(\frac{\pi b}{\lambda }\sin\theta \right)}{\frac{\pi b}{\lambda }\sin\theta }\right|$$

and $D_{2}\left(\theta \right)$ is given by:

(14) $$D_{2}\left(\theta \right)=\left|\frac{\sin\left(M\frac{\pi h}{\lambda }\sin\theta \right)}{M\sin\left(\frac{\pi h}{\lambda }\sin\theta \right)}\right|$$

In this case, the directivity function D(θ) can be seen as two parts: $D_{1}\left(\theta \right)$ is a single “cell” directivity function, only relating to the length of “cells” b; another part $D_{2}\left(\theta \right)$ is a single array element directivity function, and is related to the “inter-cell” spacing h.

**Figure 6:**
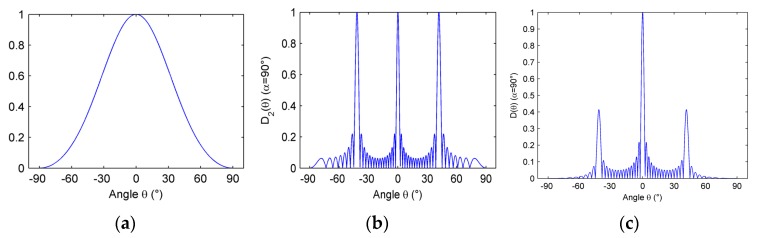
Directivity pattern of function (**a**) $D_{1}\left(\theta \right)$, (**b**) $D_{2}\left(\theta \right)$; and (**c**) D(θ) in *y-z* plane.

Figure 6 shows the acoustic beam directivity in *y*-*z* plane. Array parameters are selected such that M=16, b=λ, h=1.5λ, *f* = 3 MHz, *c* = 1500 m/s (under water).

Analyzing $D_{1}\left(\theta \right)$, we know $D_{1}\left(\theta \right)$ has no effect on the intersection of the main lobe and the θ axis; that is, the beam main lobe width only depends on the width of the main lobe of $D_{2}\left(\theta \right)$.

For $D_{2}\left(\theta \right)=0$, we have the following simultaneous equations:

(15) $$\sin\left(M\frac{\pi h}{\lambda }\sin\theta \right)=0$$

(16) $$\sin\left(\frac{\pi h}{\lambda }\sin\theta \right)\neq 0$$

Thus, the zeros position is given by:

(17) $$\theta =\sin^{-1}\left(\frac{k\lambda }{Mh}\right)$$

where

(18) $$\left|\frac{k\lambda }{Mh}\right|\leq 1,\left(k\neq mM,m\in Z\right)$$

The main lobe width can be obtained through taking k=1 and k=−1, and the result normalized by Θ can be expressed as:

(19) $$\Theta =\frac{\sin^{-1}\left(\frac{\lambda }{Mh}\right)-\sin^{-1}\left(-\frac{\lambda }{Mh}\right)}{\pi }$$

where

(20) $$\left|\pm \frac{\lambda }{Mh}\right|\leq 1$$

From Equation (19), it can be seen that the main lobes have a relationship with the “cell” number of element and the “inter-cell” spacing.

**Figure 7:**
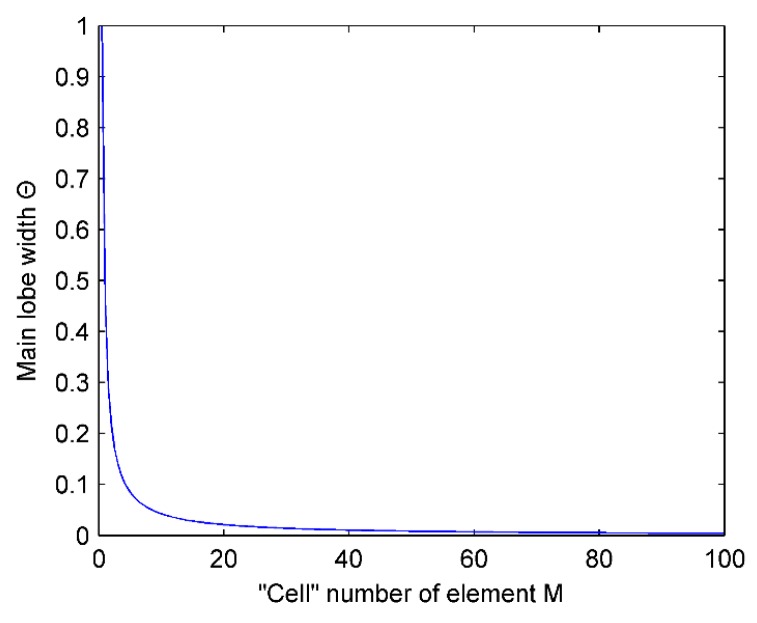
Influence of the “cell” number of element M on the width of main lobe Θ.

Figure 7 shows the relationship between the width of main lobe Θ and the “cell” number of element M. It can be observed that the width of main lobe decreases as the “cell” number of the element increases, but the trend becomes weaker. When the “cell” number of element increases from 0 to 16, the width of main lobe decreases rapidly, and when the “cell” number of element is 16 upwards, the main lobe width decreases slowly. Therefore, it is considered that, when the “cell” number of element is 16, it can obtain good sound beam directivity in the general case.

**Figure 8:**
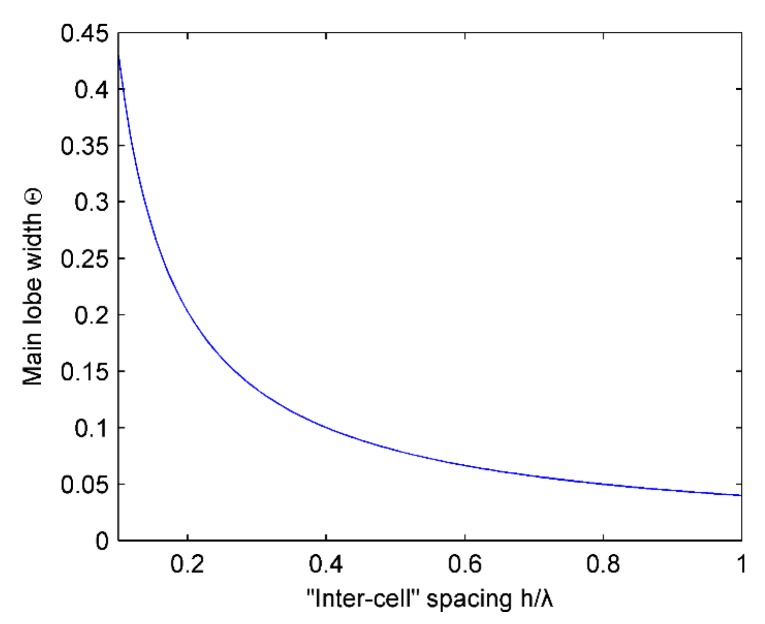
Influence of “inter-cell” spacing h on the width of main lobe Θ.

Figure 8 shows the relationship between the “inter-cell” spacing h and the width of the main lobe Θ. It can be seen that the main lobe width decreases as the ratios of h/λ increases. Under certain wavelength circumstances, the “inter-cell” spacing needs to increase to get a sharpness main lobe. However, according to later analysis, this will introduce grating lobes when the “inter-cell” spacing increase exceeds a certain range. In practical design, a consideration in compromise is needed to determine the value of the “inter-cell” spacing.

#### 4.1.2. Grating Lobe and Side Lobe

If we have the right zeros of the first grating lobe on the left side of the main lobe –90° away (considering the symmetry of the beam directivity function, it is equivalent to have the first grating lobe on the right side of the main lobe out of 90°), all the grating lobes are eliminated.

In the expression $D_{2}\left(\theta \right)$, the grating lobe and the main lobe have the same amplitude, 1. Thus, we can find the peak position of the grating lobe by letting $D_{2}\left(\theta \right)=1$. The peak position of the grating lobe is given by:

(21) $$\frac{\pi h}{\lambda }\sin\theta =n\pi$$

where n∈Z.

Thus, the expression of θ can be expressed as follows:

(22) $$\theta =\sin^{-1}\left(\frac{n\lambda }{h}\right)$$

The grating lobe is symmetric about the main lobe, so we consider the left first grating lobe only. Then the peak position of the grating lobe θ becomes:

(23) $$\theta =\sin^{-1}\left(-\frac{\lambda }{h}\right)$$

In consideration of the zeros expression (Equation (17)) of the directional function in *y-z* plane previously obtained and the peak position of the grating lobe (Equation (23)), we know that θ is the right intersection (right zeros) of the first grating lobes on the left side of the main lobe and the θ axis when k is −(M−1), that is:

(24) $$\theta =\sin^{-1}\left(-\frac{\left(M-1\right)\lambda }{Mh}\right)$$

Thus, the condition of the “inter-cell” spacing h can be obtained by:

(25) $$-\frac{\left(M-1\right)\lambda }{Mh}\leq -1$$

that is:

(26) $$h\leq h_{\text{max}}=\frac{\left(M-1\right)\lambda }{M}$$

Considering Equation (20), the “inter-cell” spacing h ranges:

(27) $$\frac{\lambda }{M}\leq h\leq \frac{\left(M-1\right)\lambda }{M}$$

From the above analysis, it can be obtained that the greater the “inter-cell” spacing h is, the better the directivity will be, but the “inter-cell” spacing h should not exceed the upper limit of $\frac{\left(M-1\right)\lambda }{M}$.

The position θ of the first side lobe peak on the left side of the main lobe can be approximately regarded as the center of the left and the right zeros of the side lobe (the *k* of Equation (17) is $-\frac{3}{2}$), that is:

(28) $$\theta_{p_{\text{max}}}=\sin^{-1}\left(-\frac{3\lambda }{2Mh}\right)$$

Because the directivity function has been normalized, the ratio of amplitude of the maximum side lobe and main lobe can be obtained by bringing Equation (28) into Equation (12).

(29) $$\xi =D\left(\theta_{p_{\text{max}}}\right)=\left|-\frac{\sin\left(\frac{3\pi }{2M}\cdot \frac{b}{\lambda }\cdot \frac{\lambda }{h}\right)}{\frac{3}{2}\pi \cdot \frac{b}{\lambda }\cdot \frac{\lambda }{h}\cdot \sin\left(\frac{3\pi }{2M}\right)}\right|$$

The effects of the “cell” number of element M, the “cell” length b and the “inter-cell” spacing h of single array element on the amplitude ratio ξ of the maximum side lobe and main lobe, respectively, are discussed below.

**Figure 9:**
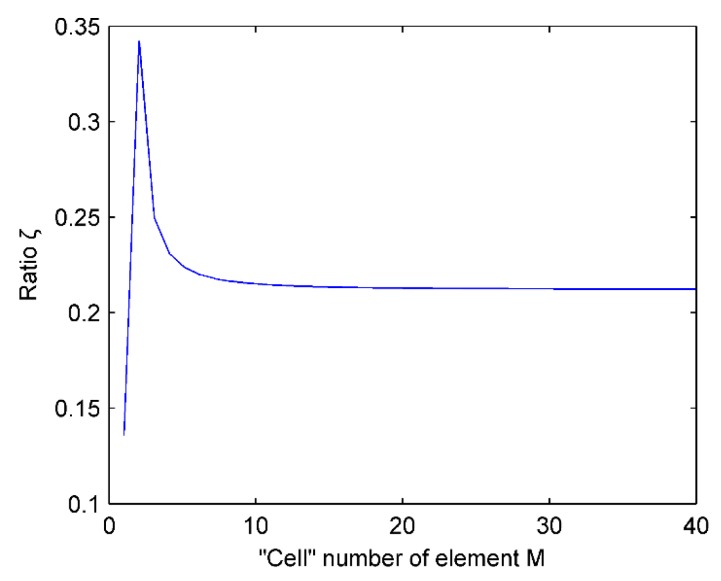
Influence of “cell” number of element on the ratio ξ.

Figure 9 shows the relationship between the ratio ξ and the “cell” number of element M. The linear array parameters are selected such that b=0.4λ, h=0.5λ, and *f* = 3 MHz. It reveals that when the “cell” number of element M is greater than 8, the ratio ξ changes slowly. For the practical MEMS linear array, in order to increase the sound radiation pressure, the “cell” number of element M meets this condition in general. Thus, we may argue that the “cell” number of element M has little effect on the ratio of the amplitude of the maximum side lobe and the main lobe.

**Figure 10:**
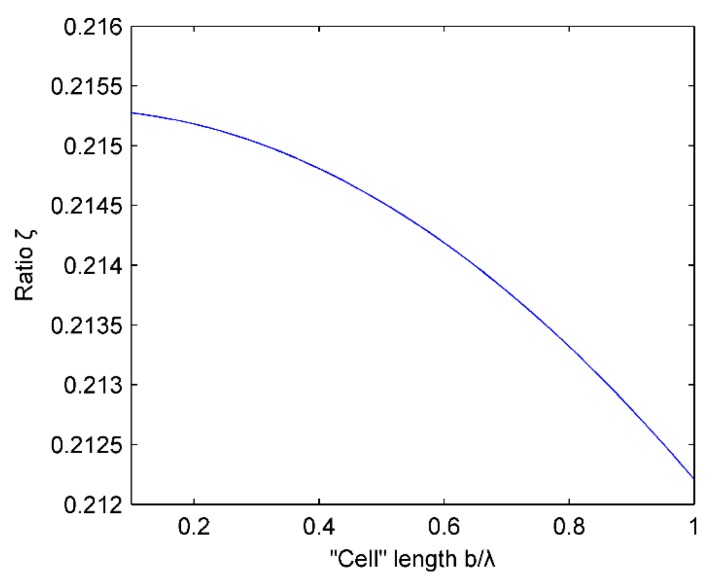
Influence of “cell” length on the ratio ξ.

Figure 10 shows the relationship between the ratio ξ and the “cell” length b. The linear array parameters are selected such that M=16 and h=λ. As can be seen from the plot, ξ is negatively correlated with the ratio of b/λ in the range (0,λ), but it decreases very slowly. Taking into account that the “cell” length b is less than the “inter-cell” spacing h, the “cell” length b has some effect on the side lobe, but it is not obvious.

**Figure 11:**
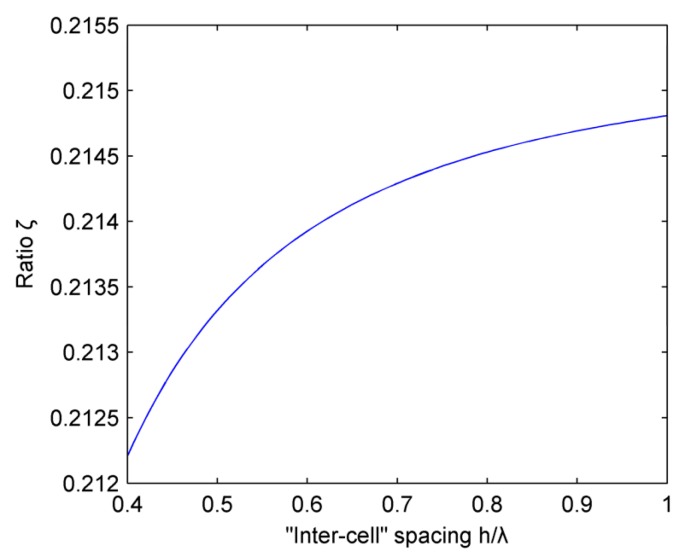
Influence of “inter-cell” spacing on the ratio ξ.

Figure 11 shows the relationship between the ratio ξ and the “inter-cell” spacing *h*. The linear array parameters are selected such that M=16 and b=0.4λ. We should consider that the “inter-cell” spacing h is greater than the “cell” length b. It could be concluded from this figure that ξ is positively correlated with the ratio of h/λ in the range (0.4λ,λ), but it increases very slowly. We can believe that the “inter-cell” spacing h has little effect on the side lobes.

### 4.2. Optimization in the Lateral Direction (x-z Plane, α = α_0_ = 0°)

The discussion of the parameters in the lateral direction is similar to that in the elevation direction, thus we do not repeat it here. The results of discussing parameters in the lateral direction are summarized as follows.

Elements number N: With the increasing of the number of array elements, the main lobe becomes narrower, and the side lobe becomes smaller. Taking into account the practical production process, it can meet good sound beam directivity when N is 16.

Steering angle $\theta_{0}$: The larger the steering angle is, the wider the main lobe will be. Without introducing the grating lobes, the smaller the steering angle is, the bigger the upper bound of the array element spacing will be. We can be appropriate to select 30° as the steering angle.

Inter-element spacing d: With the increasing of the array inter-element spacing, the main lobe becomes narrower. However, if the inter-element spacing is too large, it will introduce grating lobes. The range is:

(30) $$\frac{\lambda }{N\left(1-\sin\theta_{0}\right)}\leq d\leq \frac{\left(N-1\right)\lambda }{N\left(1+\sin\theta_{0}\right)}$$

Elements (“cell”) width a: It has little influence on the beam directivity, which is similar to the length of “cell”. Increasing the value can increase the sound radiation pressure and obtain a better signal to noise ratio.

### 4.3. Simulation Results

**Figure 12:**
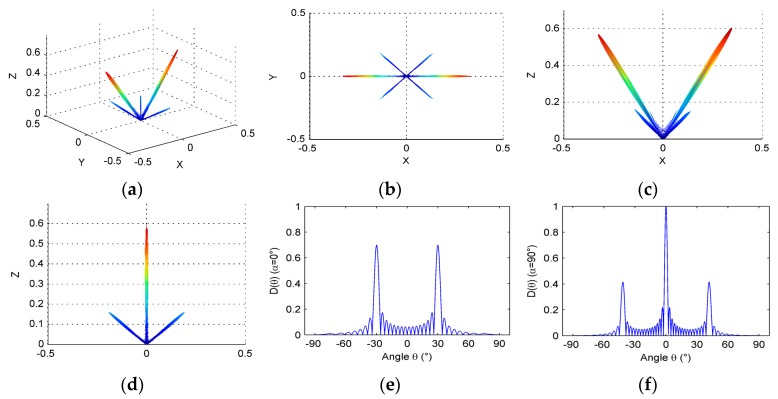
Directivity of linear phased array: (**a**) three-dimensional directivity; (**b**) directivity pattern in *x*-*y* plane; (**c**) directivity pattern in *x*-*z* plane; (**d**) directivity pattern in *y*-*z* plane; (**e**) directivity pattern when $\alpha =\alpha_{0}=0{}^{\circ}$; and (**f**) directivity pattern when $\alpha =\alpha_{0}=90{}^{\circ}$.

For linear array in Figure 4, if it is not optimized, it cannot achieve good sound directivity in general. In addition to a wide main lobe in the steering angle direction, it will be accompanied by a large grating lobes and high side lobes. Taking an example of no optimization array, the linear array directivity is shown as Figure 12. The linear array parameters are selected such that N=16, a=0.9λ, d=λ, M=16, b=λ, h=1.5λ, $\theta_{0}=30{}^{\circ}$, and *f* = 3 MHz. From Figure 12c, it is obvious that a great grating lobe exists outside the direction of steering angle. The main lobe has serious energy “leakage” in the elevation direction, as shown in Figure 12d. Figure 12a,b also confirms the above point.

**Figure 13:**
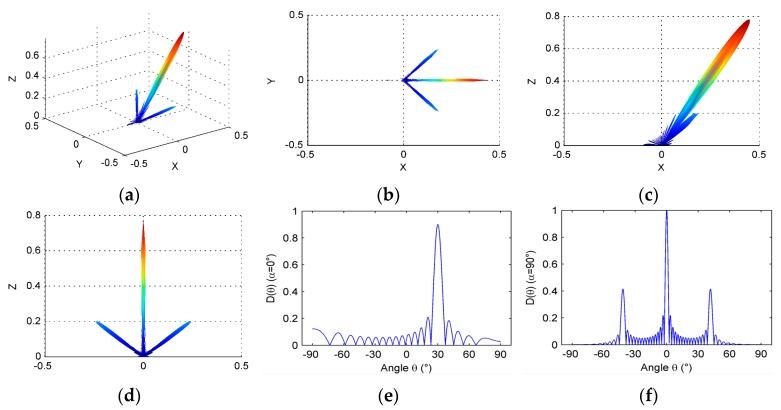
Directivity of linear phased array after optimizing parameters in the lateral direction of the array: (**a**) three-dimensional directivity; (**b**) directivity pattern in *x*-*y* plane; (**c**) directivity pattern in *x*-*z* plane; (**d**) directivity pattern in *y*-*z* plane; (**e**) directivity pattern when $\alpha =\alpha_{0}=0{}^{\circ}$; and (**f**) directivity pattern when $\alpha =\alpha_{0}=90{}^{\circ}$.

If the optimization of the linear array is not considered in the elevation direction (that is, only optimizing the lateral direction parameters), the following array parameters are selected such that N=16, a=0.5λ, d=0.6λ, M=16, b=λ, h=1.5λ, $\theta_{0}=30{}^{\circ}$, and *f* = 3 MHz. Optimized beam directivity is shown below (see Figure 13).

From Figure 13c,e, we can find out that after optimizing the lateral direction parameters, the grating lobes have been eliminated in this direction. However, in Figure 13d, there are still serious grating lobes in the elevation direction, and the side lobes also need to be optimized.

**Figure 14:**
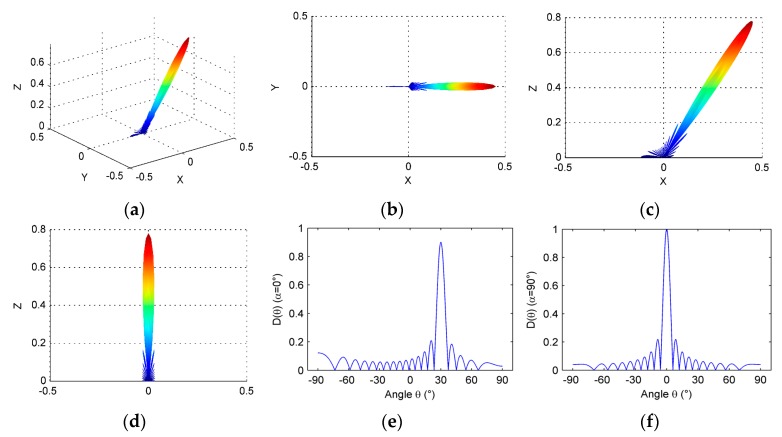
Directivity of linear phased array after optimizing parameters comprehensively: (**a**) three-dimensional directivity; (**b**) directivity pattern in *x*-*y* plane; (**c**) directivity pattern in *x*-*z* plane; (**d**) directivity pattern in *y*-*z* plane; (**e**) directivity pattern when $\alpha =\alpha_{0}=0{}^{\circ}$; and (**f**) directivity pattern when $\alpha =\alpha_{0}=90{}^{\circ}$.

At the same time, the elevation direction is considered, and the following optimized parameters are used in simulation: N=16, a=0.5λ, d=0.6λ, M=16, b=0.5λ, h=0.6λ, $\theta_{0}=30{}^{\circ}$, and *f* = 3 MHz. Optimized beam directivity is shown below (see Figure 14).

In Figure 14, the beam focusing only exists as a maximum value in the direction of the steering angle, 30°, and it can be considered that the optimized array obtained a good beam directivity, whose beam focusing has a narrow main lobe, low side lobes, and no grating lobes.

It is worth pointing out that compared with Figure 13 and Figure 14, we can strongly find out that the grating lobes around the main lobe in the elevation direction is eliminated. In other words, it can suppress the energy leaking into non-azimuthal directions if the elevation dimension is taken into account. The results are consistent with the Wooh’s work [18] and show sideleaking energy is mainly resulted from grating lobes in non-azimuthal plane. 

## 5. Conclusions

In order to increase radiation sound pressure of the MEMS linear phased array working in bending vibration mode at high frequency, the structure based on “multi-cell” element is designed. The theoretical analyses of the dynamic equation of a fixed rectangular composite plate and ANSYS simulation are used to more accurately predict the array resonant frequency. According to the Huygens principle and product theorem, the linear array directivity function is obtained. The effects of the correlation parameters of the array on the three-dimensional sound beam directivity are discussed in two directions, the lateral and elevation direction. The results show that the width of the array element (the width of “cell”) has no effect on the main lobe, and has little effect on the side lobes. The increasing of inter-element spacing and the “inter-cell” spacing can make the main lobe narrower. However, both of them have an upper limit in order to eliminate grating lobes. The increasing of the number of elements and the “cell” number of element can also make the main lobe narrower. Thus, the beam directivity of linear array is determined by several parameters simultaneously. The simulation results show that optimizing the linear array parameters, both in the lateral and elevation directions, can greatly improve the three-dimensional beam focusing for MEMS linear phased array, which is obviously different from the traditional linear array.
